# Coping Strategies, Pain, and Quality of Life in Patients with Breast Cancer

**DOI:** 10.3390/jcm10194469

**Published:** 2021-09-28

**Authors:** Edyta Ośmiałowska, Weronika Misiąg, Mariusz Chabowski, Beata Jankowska-Polańska

**Affiliations:** 1 Department of Clinical Nursing, Faculty of Health Science, Wroclaw Medical University, 51-618 Wroclaw, Poland; edziaosmial@wp.pl (E.O.); bianko@poczta.onet.pl (B.J.-P.); 2 Student Research Group No 180, Faculty of Medicine, Wroclaw Medical University, 50-367 Wroclaw, Poland; weronika.misiag@student.umed.wroc.pl; 3 Department of Clinical Nursing, Division of Oncology and Palliative Care, Faculty of Health Science, Wroclaw Medical University, 51-618 Wroclaw, Poland; 4 Department of Surgery, 4th Military Teaching Hospital, 50-981 Wroclaw, Poland

**Keywords:** pain, quality of life, cancer coping strategy, Mini-MAC, EORTC QLQ–C30

## Abstract

Introduction: Each year, around 16,500 women in Poland are diagnosed with breast cancer, the second most common cause of death in women. In Poland, nearly 70,000 women live with breast cancer diagnosed within the last 5 years. Quality of life (QoL) research is particularly important in cancer patients, as it provides knowledge on their psychological and physical health, as well as the environment in which the patients function, all of which is essential to implementing multidisciplinary care involving the best use of the appropriate methods. Carrying the burden of cancer is a major challenge for patients. The strategy that patients use to cope with breast cancer significantly affects their quality of life. The purpose of the study is to assess the impact of coping strategies on the QoL in breast cancer patients. Material and Methods: The prospective study included a group of 202 women who had undergone surgical treatment for breast cancer at the Lower Silesian Cancer Center and who reported for follow-up appointments at the Oncology Clinic and the Surgical Oncology Clinic. For the study, we used the: EORTC QLQ-C30 cancer questionnaire, EORTC QLQ-BR23 module, Mental Adjustment to Cancer (Mini-MAC) scale, visual analog scale (VAS) for pain intensity, as well as the patients’ medical records, hospital records, and our own survey form. Results: The mean patient age was 53 years. Most patients had been diagnosed with cancer between one and two years before. In the women studied, there was a negative association between QoL and the choice of a destructive strategy for coping with cancer, and a positive one between QoL and a constructive coping strategy. Severe pain caused by the disease and its treatment significantly decreased the patients’ QoL in multiple domains. Conclusions: Patients choosing constructive strategies obtained higher QoL scores, while greater reliance on destructive coping strategies was associated with significantly worse QoL. In all functioning domains, higher levels of pain were associated with poorer QoL and more severe symptoms associated with the disease and its treatment.

## 1. Introduction

Each year, approximately 16,500 women in Poland [1] are diagnosed with breast cancer, the second most common cause of death in women [2]. In Poland, nearly 70,000 women live with breast cancer diagnosed within the last five years. The mean age at diagnosis is 61 years, but younger women are being increasingly often diagnosed with this type of cancer. Most deaths from breast cancer occur in women above 50 years of age (90%), and the risk of death increases with age [3]. The Polish National Cancer Registry reports that the incidence of breast cancer doubled in the last 30 years. In the year 2000, 11,853 patients were diagnosed with breast cancer, in 2010—more than 15,700, and in 2013—17,142. The greatest increase in the risk of developing breast cancer has been noted in women aged 45–69 [4,5].

In cancer patients, quality of life (QoL) research is particularly important, as it enables a more comprehensive assessment and provides more insight into the patients’ psychological and physical health, as well as the environment in which they function. This additional information on patients’ QoL is essential to implementing multidisciplinary care involving the best use of the appropriate methods. QoL is shaped by socio-demographic factors, clinical factors including pain, and psycho-social factors such as acceptance of illness, strategies for coping with cancer, and strategies for coping with pain and stress. Determinants of QoL in women with breast cancer include pain, limitations in functioning, difficulties in daily activities, anxiety, and depression. Higher levels of anxiety are associated with greater feelings of hopelessness, more adverse effects of treatment after breast surgery, poorer body image, and poorer sexual functioning. An important aspect of QoL is the choice of the operating method. Breast reconstruction increases the esthetic satisfaction, which improves the patient well-being and QoL.

Pain caused by cancer can take two forms: physical and psychological. Pain severity is significantly correlated with physical, social, cognitive, emotional, and sexual functioning, body image, and future outlook.

Carrying the burden of cancer is a major challenge for patients. The strategy that patients use to cope with their breast cancer significantly affects their quality of life. A number of strategies for coping with cancer have been identified, including: fighting spirit, positive redefinition, helplessness/hopelessness, and anxious preoccupation.

The purpose of the study is to assess the impact of coping strategies on the QoL in breast cancer patients.

## 2. Materials and Methods

The prospective study included a group of 250 women who had undergone surgical treatment for breast cancer at the Lower Silesian Cancer Center and who reported for follow-up appointments at the Oncology Clinic and the Surgical Oncology Clinic. Forty-five patients dropped out during the study, either due to poor health or to a negative attitude toward the treatment. Moreover, three patients were excluded from the study due to the diagnosis of another cancer. There were no exclusions for severe depression or comorbidities. The following standardized instruments (validated in the Polish population) were used: The EORTC QLQ-C30 cancer questionnaire, the EORTC QLQ-BR23 breast-cancer-specific module, the Mental Adjustment to Cancer (Mini-MAC) scale, a visual analog scale (VAS) for pain intensity, as well as the patients’ medical records, hospital records, and the study’s own survey form assessing the patients’ clinical and socio-demographic situation.

The mean patient age was 53 (SD ± 10.3 years), the youngest patient was 26 years old, and the oldest was 75 years old. Most patients had been diagnosed with cancer between 1 and 2 years before the study (52.4%). The 22.8% of patients had been suffering from cancer for 2–5 years and 24.8% for over 5 years. Most patients had completed higher education (39.6%), were professionally active (63.9%), lived in urban areas (77.2%), and reported their financial standing and living conditions as good (63.4%). Single and childless women were a minority (24.3% and 14.9%, respectively).

In 53% of the surveyed patients, the disease was diagnosed during breast self-examination. As a result of screening mammography and during a visit to a doctor, respectively 25.7% and 21.3% of patients were diagnosed. In addition, 37.6% of the surveyed women underwent partial breast excision, 35.1% had a total amputation, and in 26.7% of the surveyed women breast amputation with simultaneous breast reconstruction were performed.

Inclusion criteria were: age between 18 and 75 years, diagnosis of early invasive breast cancer, primary surgical treatment with adjuvant chemotherapy or radiation therapy, and voluntary consent to participate. Exclusion criteria were: clinically advanced cancer (stage IV with metastasis), non-surgical treatment, another concurrent cancer or another severe comorbidity (measured as the presence or absence), and severe depression requiring specialist treatment.

The study was approved on 24 April 2018 by the Medical University Bioethics Committee (approval no. KB–196/2018).

The European Organization for Research and Treatment of Cancer (EORTC) QLQ-C30 questionnaire on quality of life comprises 30 items for the assessment of the patient’s physical (5 items), role (2 items), emotional (4 items), cognitive (2 items), and social functioning (2 items). With 3 symptom scales and 6 individual items, the questionnaire also assesses fatigue, nausea and vomiting, pain, dyspnea, insomnia, appetite loss, constipation, diarrhea, and financial difficulties caused by the disease [6].

The EORTC QLQ-BR23 module provides a more accurate assessment of QoL in patients with breast cancer. It comprises 5 multi-item scales concerning body image, sexual functioning, treatment side effects, and breast- and arm-related symptoms. It also asks questions on the patient’s sexual enjoyment as well as their concerns about the prognosis and hair loss. The listed scales from both questionnaires represent a score range of 0–100 points. Greater intensity of a given characteristic corresponds to a higher score. 

The Mental Adjustment to Cancer (Mini-MAC) scale evaluates the ways in which the patient copes with cancer. It identifies constructive and destructive strategies, including fighting spirit, positive redefinition, helplessness/hopelessness, and anxious preoccupation. Each strategy is rated using 7 statements. Scores in each category range between 7 and 28 points, and higher scores indicate a greater intensity of behaviors associated with a particular strategy [7].

A visual analogue scale (VAS) is an instrument comprising a numerical scale representing pain severity, with 0 standing for no pain at all, and 10 for unbearable pain. The self-designed questionnaire, the patients’ medical records, and hospital records allowed for assessing the age, education level, financial standing, professional activity, place of residence, stable relationship, and having children (Table 1) as well as the assessment of the clinical characteristics: the time of the diagnosis, the method of detection, and the type of surgery.

Statistical analysis results for qualitative variables are shown in contingency tables as numbers (*n*) and percentages (%), with the chi-squared test used to assess the strength of association between two variables. For quantitative variables, distribution normality was verified using the Kolmogorov–Smirnov or Shapiro–Wilk test. Means for quantitative variables in multiple groups were compared using a single-factor analysis of variance (ANOVA). All analyses used a significance threshold of *p* < 0.05, and Tukey’s LSD test was used to perform the analyses.

## 3. Results

Comparative analysis of QoL in the sexual functioning (BRSEF) and sexual enjoyment (BRSEE) domains demonstrated associations with some socio-demographic variables (*p* < 0.05), listed in Table 2 and Table 3.

Analysis of mental adaptation to cancer based on Mini-MAC scores showed that the most common coping strategies in the cancer patients studied were fighting spirit and positive redefinition (Table 4). Anxious preoccupation and helplessness/hopelessness were the least common. The vast majority preferred the constructive coping style (22.8 ± 2.7, or standard ten (sten) 7.0 ± 1.4) to the destructive coping style (14.3 ± 4.1, or sten score 3.6 ± 2.0).

Our analysis of general QoL based on the EORTC QLQ-C30 questionnaire in the group with a constructive coping strategy (identified by the Mini-MAC) only demonstrated a statistically significant relationship in terms of role functioning (Table 5). High scores for constructive coping were associated with an RF score of 23.3 ± 19.0, compared to 17.1 ± 22.0 for low ones (*p* = 0.047). In turn, in the analysis of relationships between QoL measured by the EORTC QLQ-BR23 questionnaire and the constructive coping strategy identified by the Mini-MAC, a significant relationship was only found with the “arm-related symptoms” domain (Table 6).

When associations between QoL (EORTC QLQ-C30) and the destructive coping strategy (Mini-MAC) were analyzed, significant correlations were found in all functioning domains. Higher scores for the destructive strategy were associated with poorer QoL. Analysis results are shown in Table 7, with significant associations identified at *p* < 0.001. Similarly to EORTC QLQ-C30, EORTC QLQ-BR23 scores were also correlated with scores for the destructive coping strategy (Table 8). The most significant changes in QoL were found in the body image, sexual functioning, and future outlook domains (*p* < 0.001). The choice of this strategy was also associated with more severe disease symptoms and treatment adverse effects, as shown in Table 8.

To analyze the relationship between QoL and pain levels, comparative analysis of EORTC QLQ-C30 and VAS scores was performed. Higher pain levels were associated with poorer perceived QoL in the following domains: general health, physical functioning, role functioning, emotional functioning, cognitive functioning, and social functioning (*p* < 0.001) (Table 9).

Comparative analysis of EORTC QLQ-BR23 and VAS scores demonstrated better functioning in patients with mild or moderate pain, compared to those experiencing severe pain, in terms of: body image (*p* < 0.001), sexual functioning (*p* < 0.001); no statistically significant association was found in terms of sexual enjoyment (*p* = 0.021) or future outlook (*p* = 0.023) (Table 10). More severe symptoms in most EORTC QLQ-BR23 domains were also observed in patients with high VAS pain scores (Table 10).

## 4. Discussion

Each year, breast cancer contributes to the death of many women. Early diagnosis and advanced diagnostics allow for reducing mortality from breast cancer [8,9,10,11]. Modern treatment relies on combination therapy involving surgery, chemotherapy, and radiation therapy. Unfortunately, cancer treatment may produce a number of adverse effects with a patient-specific incidence and severity. Many women perceive the surgical treatment as disfiguring and leading to a loss of femininity [12,13]. The cosmetic effect and intense pain (especially in the arm and breast) contribute to poorer daily functioning and interfere with professional activity, which is reflected in the patients’ QoL [14]. Research demonstrates the better outcomes of breast reconstruction compared to a mastectomy [15]. The choice of this high safety profile method is strongly associated with an improvement in patient esthetic satisfaction and QoL [16]. Good cosmetic outcome scores have a significant correlation with psychological adjustment [17,18]. In a study by Brunault et al. [19], QoL was dependent on psychological and patient-specific factors (age, depression, coping with stress) more than on biological ones (treatment type, cancer stage).

Carrying the burden of cancer is a major challenge for patients. The strategy that patients use to cope with their breast cancer significantly affects their quality of life. A number of strategies for coping with cancer have been identified, including: fighting spirit, positive redefinition, helplessness/hopelessness, and anxious preoccupation. A patient taking the constructive approach is motivated to treat their illness as a challenge and undertake action to combat it. Positive redefinition allows the patient to find hope and satisfaction in life, while maintaining full awareness of the severity of their illness; meanwhile, destructive strategies manifest in feelings of powerlessness, anxiety, and a tendency to interpret any symptom as a sign of health deterioration. The latter may exacerbate the adverse effects of mastectomy, in particular the arm- and breast-related symptoms, and promote a passive approach to the illness. In published literature, the choice of an active and constructive strategy not only improved QoL but also contributed to longer survival [20,21,22].

T. Kershaw et al. [23] demonstrated that both constructive and destructive strategies for coping with cancer significantly affected QoL. Their findings suggest a stronger impact of the chosen coping strategy on the psychological QoL aspects than on physical ones. Notably, negative strategies have a strong impact on patients’ lives and impair their daily functioning considerably, while a positive approach to coping with cancer does not improve QoL as strongly. In literature, Chabowski et al. documented the complex relationship between QoL and coping with disease [24]. Moreover, multiple studies show that one’s choice of coping strategy may depend on the stage of treatment and time from diagnosis [25]. The longer the time from diagnosis, the less likely patients are to adopt positive strategies, while no change over time was observed for the “helplessness” and “anxious preoccupation” strategies [26].

Our study corroborates findings from other research papers, showing the patients’ ability to use constructive coping strategies had statistically significant associations with QoL in all functioning domains, as well as with symptom severity and adverse effects of treatment. An important domain affected by an inability to positively cope with the disease, as well as by severe pain, involved sexual functioning and sexual enjoyment. Our study identified a statistically significant link with these domains. This was attributable both to disease symptoms, adverse effects of treatment, or severe stress, and to poorer QoL, e.g., due to depression. Women who had undergone mastectomy did not feel attractive, and their sexuality—an integral, fundamental, natural part of human personality—was affected [27,28]. Sexuality is a crucial factor in QoL, as well as a fundamental bio-socio-psychological contributor to the development of personality, temperament, and enjoyable life experiences; a positive experience of sexuality facilitates coping with cancer, which is why the aspect is relevant to clinicians [29,30,31].

There is no doubt that severe pain, especially chronic, does reduce QoL. Notably, patients experiencing intense pain rated their QoL much lower in the physical, emotional, social, sexual, and cognitive domains [32]. This also affected their functioning in their social roles. Similar conclusions in this respect come from the present study and from that by Caffo et al. [33]. Notably, patients who experienced mild or no pain obtained much higher QoL scores. Pain after mastectomy can make patients anxious about recurrence, which also affects QoL [33].

An awareness of women’s individual needs, coping strategies, and the impact of pain on their emotional and physical QoL can help make the work of healthcare professionals more effective and provide women with comprehensive care and support they need in the difficult process of cancer treatment.

## 5. Conclusions

Patients choosing constructive strategies obtained higher QoL scores, while greater reliance on destructive coping strategies was associated with significantly lower QoL.

In all functioning domains, higher levels of pain were associated with poorer QoL and more severe symptoms associated with the disease and its treatment.

## Figures and Tables

**Table 1:** General sociodemographic characteristics of the studied patients.

Variable	*n* (%)
1. Number of respondents	202 (100%)
2. Age (years)	
*M* ± *SD*	53.0 ± 10.3
*Me* (*Q*_1_; *Q*_3_)	52 (45; 61)
Range	26–75
3. Education	
Primary	9 (4.5%)
Vocational	35 (17.3%)
High school	78 (38.6%)
College/university	80 (39.6%)
4. Financial standing	
Very good	36 (17.8%)
Good	128 (63.4%)
Unsatisfactory	35 (17.3%)
Poor	3 (1.5%)
5. Professional activity	
Yes	129 (63.9%)
No	73 (36.1%)
6. Residence	
Urban	156 (77.2%)
Rural	46 (22.8%)
7. In a stable relationship	
Yes	153 (75.7%)
No	49 (24.3%)
8. Children	
Yes	172 (85.1%)
No	30 (14.9%)
9. Number of children	
0	39 (19.3%)
1	72 (35.6%)
2	76 (37.6%)
3 and more	15 (7.4%)

*M*—mean; *SD*—standard deviation; *Me*—median; *Q*_1_—lower quartile; *Q*_3_—upper quartile; *n*—number; %—percentage.

**Table 2:** Sexual functioning (EORTC QLQ-BR23 BRSEF domain) in patients differing in terms of selected socio-demographic and clinical characteristics, with test results.

Characteristic (Variable)	BRSEF	Test Result *p*
	*M ± SD*	
1. Age (years)		0.179
2. Education		0.980
3. Financial standing		0.054
4. Professional activity		0.628
5. Residence		0.006
Urban	47.8 ± 32.7	
Rural	33.8 ± 29.2	
6. In a stable relationship		0.312
7. Has children		0.179
8. Duration of illness (years)		0.084
9. Disease diagnosed during a doctor’s visit:		0.042
No	45.3 ± 34.4	
Yes	34.7 ± 29.1	
10. Chronic illness		0.064
11. Satisfied with treatment:		0.001
Yes	61.7 ± 23.6	
Yes, moderately	38.9 ± 25.7	
Rather not	37.9 ± 29.0	
No	32.5 ± 30.9	
12. Time from the procedure		0.464
13. Cosmetic result:		<0.001
Very good	55.0 ± 26.1	
Good	53.4 ± 25.7	
Poor	37.0 ± 30.0	
Very poor	26.3 ± 30.0	
14. Negative impact of the surgery on personal life:		<0.001
Yes, major impact	18.9 ± 20.4	
Yes, minor impact	31.2 ± 30.7	
No impact	50.3 ± 29.1	

**Table 3:** Sexual enjoyment (EORTC QLQ-BR23 BRSEE domain) in patients differing in terms of selected socio-demographic and clinical characteristics, with test results.

Characteristic (Variable)	BRSEE	Test Result *p*
	*M ± SD*	
1. Age (years)		0.022
46 or earlier	49.6 ± 37.4	
47–59	39.8 ± 33.9	
60 or more	29.2 ± 34.5	
2. Education		0.025
Primary or vocational	27.4 ± 32.3	
High school	38.8 ± 34.0	
College/university	46.9 ± 37.4	
3. Financial standing		<0.001
Very good	52.9 ± 34.0	
Good	42.9 ± 35.6	
Unsatisfactory or poor	14.0 ± 24.0	
4. Professional activity		0.005
No	28.6 ± 34.5	
Yes	44.8 ± 35.1	
5. Residence		0.038
Urban	42.5 ± 36.8	
Rural	29.3 ± 30.0	
6. In a stable relationship		0.001
No	21.9 ± 35.2	
Yes	43.9 ± 34.4	
7. Has children		0.137
8. Duration of illness (years)		0.995
9. Disease diagnosed during a doctor’s visit		0.176
10. Comorbidities		0.009
No	41.2 ± 35.6	
Yes	12.1 ± 22.5	
11. Satisfaction with treatment		0.099
12. Time from the procedure		0.358
13. Cosmetic result		0.077
14. Negative impact of the surgery on personal life		0.049
Yes, major impact	33.3 ± 34.0	
Yes, minor impact	39.0 ± 34.9	
No impact	49.6 ± 37.4	

**Table 4:** Mini-MAC questionnaire scores.

Cancer Coping Strategy	Results (*n*)	
Anxious preoccupation (points)		
*M* ± *SD*	16.6 ± 4.5	
*Me* (*Q*_1_; *Q*_3_)	17 (13; 20)	
*Min*–*Max*	7–28	
Fighting spirit (points)		
*M* ± *SD*	23.3 ± 3.4	
*Me* (*Q*_1_; *Q*_3_)	24 (21; 26)	
*Min*–*Max*	4–28	
Helplessness/hopelessness (points)		
*M* ± *SD*	11.9 ± 4.4	
*Me* (*Q*_1_; *Q*_3_)	11 (9; 14)	
*Min*–*Max*	6–25	
Positive redefinition (points)		
*M* ± *SD*	22.3 ± 3.1	
*Me* (*Q*_1_; *Q*_3_)	23 (21; 25)	
*Min*–*Max*	7–28	
Constructive style (points)		
*M* ± *SD*	22.8 ± 2.7	
*Me* (*Q*_1_; *Q*_3_)	24 (22; 25)	
*Min*–*Max*	16–28	
Destructive style (points)		
*M* ± *SD*	14.3 ± 4.1	
*Me* (*Q*_1_; *Q*_3_)	14 (11; 17)	
*Min*–*Max*	7–25	
Constructive style (sten)		
*M* ± *SD*	7.0 ± 1.4	
*Me* (*Q*_1_; *Q*_3_)	7 (6; 8)	
*Min*–*Max*	4–10	
Destructive style (sten)		
*M* ± *SD*	3.6 ± 2.0	
*Me* (*Q*_1_; *Q*_3_)	4 (2; 5)	
*Min*–*Max*	1–8	
Constructive style	*n*	%
Low score	5	2.5
Moderate score	69	34.2
High score	128	63.4
Destructive style		
Low score	126	62.4
Moderate score	59	29.2
High score	17	8.4

*M*—mean; *SD*—standard deviation; *Me*—median; *Q*_1_—lower quartile; *Q*_3_—upper quartile; *Min*—lowest value; *Max*—highest value; *n*—number; %—percentage.

**Table 5:** Quality of life (EORTC QLQ-30C scores) in patients differing in terms of scores for the constructive coping strategy (Mini-MAC), with analysis of variance results.

QoL Evaluation—EORTC QLQ-30C	Constructive Coping Strategy (Mini-MAC)			ANOVA *p*
	Low Score Sten 0–4	Moderate Score Sten 5–6	High Score Sten 7–10	
	*n* = 5	*n* = 69	*n* = 128	
General health (QL)				0.103
*M* ± *SD*	45.0 ± 23.3	55.2 ± 18.9	60.1 ± 21.3	
*Me* (*Q*_1_; *Q*_3_)	33 (33; 50)	58 (42; 67)	67 (50; 75)	
*Min*–*Max*	25–83	0–100	0–100	
Physical functioning (PF)				0.163
*M* ± *SD*	23.8 ± 17.2	23.5 ± 17.2	38.7 ± 20.8	
*Me* (*Q*_1_; *Q*_3_)	20 (13; 27)	20 (13; 33)	40 (33; 53)	
*Min*–*Max*	0–87	0–73	7–60	
Role functioning (RF)				0.047
*M* ± *SD*	17.1 ± 22.0	24.9 ± 19.9	23.3 ± 19.0	
*Me* (*Q*_1_; *Q*_3_)	17 (0; 17)	17 (17; 33)	17 (17; 33)	
*Min*–*Max*	0–100	0–67	0–50	
Emotional functioning (EF)				0.097
*M* ± *SD*	35.9 ± 23.5	39.5 ± 24.8	58.3 ± 36.3	
*Me* (*Q*_1_; *Q*_3_)	33 (17; 50)	33 (25; 58)	67 (50; 83)	
*Min*–*Max*	0–100	0–92	0–92	
Cognitive functioning (CF)				0.938
*M* ± *SD*	23.3 ± 22.4	25.6 ± 23.8	24.3 ± 25.9	
*Me* (*Q*_1_; *Q*_3_)	33 (0; 33)	17 (0; 33)	17 (0; 33)	
*Min*–*Max*	0–50	0–83	0–100	
Social functioning (SF)				0.243
*M* ± *SD*	27.6 ± 22.1	31.4 ± 27.3	43.3 ± 27.9	
*Me* (*Q*_1_; *Q*_3_)	33 (17; 33)	33 (17; 50)	50 (33; 67)	
*Min*–*Max*	0–100	0–100	0–67	
Fatigue (FA)				0.054
*M* ± *SD*	57.8 ± 28.8	42.7 ± 21.8	37.6 ± 21.5	
*Me* (*Q*_1_; *Q*_3_)	67 (56; 67)	33 (33; 56)	33 (22; 56)	
*Min*–*Max*	11–89	0–100	0–100	
Nausea and vomiting (NV)				0.110
*M* ± *SD*	36.7 ± 27.4	16.4 ± 25.6	13.0 ± 25.9	
*Me* (*Q*_1_; *Q*_3_)	50 (17; 50)	0 (0; 33)	0 (0; 17)	
*Min*–*Max*	0–67	0–100	0–100	
Pain (PA)				0.102
*M* ± *SD*	46.7 ± 27.4	31.2 ± 25.6	26.0 ± 24.7	
*Me* (*Q*_1_; *Q*_3_)	50 (50; 67)	33 (0; 50)	17 (0; 33)	
*Min*–*Max*	0–67	0–100	0–100	
Dyspnea (DY)				0.787
*M* ± *SD*	13.3 ± 18.3	16.2 ± 24.8	13.5 ± 26.3	
*Me* (*Q*_1_; *Q*_3_)	0 (0; 33)	0 (0; 33)	0 (0; 8)	
*Min*–*Max*	0–33	0–100	0–100	
Insomnia (SL)				0.468
*M* ± *SD*	53.3 ± 38.0	37.3 ± 33.8	41.7 ± 33.2	
*Me* (*Q*_1_; *Q*_3_)	67 (33; 67)	33 (0; 67)	33 (0; 67)	
*Min*–*Max*	0–100	0–100	0–100	
Appetite loss (AP)				0.102
*M* ± *SD*	46.7 ± 38.0	21.3 ± 25.5	19.5 ± 28.5	
*Me* (*Q*_1_; *Q*_3_)	33 (33; 67)	0 (0; 33)	0 (0; 33)	
*Min*–*Max*	0–100	0–100	0–100	
Constipation (CO)				0.866
*M* ± *SD*	26.7 ± 27.9	20.8 ± 25.6	20.6 ± 24.4	
*Me* (*Q*_1_; *Q*_3_)	33 (0; 33)	0 (0; 33)	0 (0; 33)	
*Min*–*Max*	0–67	0–100	0–100	
Diarrhea (DI)				0.248
*M* ± *SD*	26.7 ± 43.5	9.2 ± 24.2	11.2 ± 21.0	
*Me* (*Q*_1_; *Q*_3_)	0 (0; 33)	0 (0; 0)	0 (0; 33)	
*Min*–*Max*	0–100	0–100	0–100	
Financial difficulties (FI)				0.722
*M* ± *SD*	20.0 ± 18.3	29.4 ± 31.3	30.7 ± 29.8	
*Me* (*Q*_1_; *Q*_3_)	33 (0; 33)	33 (0; 33)	33 (0; 33)	
*Min*–*Max*	0–33	0–100	0–100	

*M*—mean; *SD*—standard deviation; *Me*—median; *Q*_1_—lower quartile; *Q*_3_—upper quartile; *Min*—lowest value; *Max*—highest value.

**Table 6:** Quality of life (EORTC QLQ-BR23 scores) in patients differing in terms of scores for the constructive coping strategy, with analysis of variance results.

Functioning Scales QLQ-BR23	Constructive Coping Strategy (Mini-MAC)			ANOVA *p*
	Low Score Sten 0–4	Moderate Score Sten 5–6	High Score Sten 7–10	
	*n* = 5	*n* = 69	*n* = 128	
Body image (BRBI)				0.743
*M* ± *SD*	36.7 ± 24.7	37.1 ± 26.3	40.2 ± 29.1	
*Me* (*Q*_1_; *Q*_3_)	33 (33; 50)	33 (17; 58)	33 (17; 58)	
*Min*–*Max*	0–67	0–100	0–100	
Sexual functioning (BRSEF)				0.842
*M* ± *SD*	33.3 ± 23.6	35.5 ± 28.7	37.9 ± 31.8	
*Me* (*Q*_1_; *Q*_3_)	33 (33; 33)	33 (17; 50)	33 (0; 67)	
*Min*–*Max*	0–67	0–100	0–100	
Sexual enjoyment (BRSEE)				0.640
*M* ± *SD*	16.7 ± 23.5	38.4 ± 37.6	40.1 ± 34.7	
*Me* (*Q*_1_; *Q*_3_)	17 (8; 25)	33 (0; 67)	33 (0; 67)	
*Min*–*Max*	0–33	0–100	0–100	
Future outlook (BRFU)				0.398
*M* ± *SD*	73.3 ± 36.5	68.1 ± 31.5	74.3 ± 29.5	
*Me* (*Q*_1_; *Q*_3_)	100 (33; 100)	67 (33;100)	100(33;100)	
*Min*–*Max*	33–100	0–100	0–100	
Systemic treatment adverse effects (BRST)				0.061
*M* ± *SD*	45.3 ± 14.7	26.0 ± 17.6	30.4 ± 20.8	
*Me* (*Q*_1_; *Q*_3_)	52 (33; 52)	24 (14; 38)	29 (14; 43)	
*Min*–*Max*	27–62	0–62	0–100	
Breast-related symptoms (BRBS)				0.190
*M* ± *SD*	46.7 ± 33.6	25.6 ± 21.7	28.3 ± 26.6	
*Me* (*Q*_1_; *Q*_3_)	58 (25; 67)	25 (8; 42)	25 (8; 50)	
*Min*–*Max*	0–83	0–75	0–92	
Arm-related symptoms (BRAS)				0.038
*M* ± *SD*	38.7 ± 25.3	29.5 ± 21.5	33.3 ± 24.9	
*Me* (*Q*_1_; *Q*_3_)	33 (19; 56)	22 (11; 44)	33 (22; 44)	
*Min*–*Max*	0–100	0–89	0–67	
Concern about hair loss (BRHL)				0.076
*M* ± *SD*	41.7 ± 50.0	45.2 ± 31.1	60.1 ± 35.7	
*Me* (*Q*_1_; *Q*_3_)	33 (0; 75)	33 (33; 67)	67 (33; 100)	
*Min*–*Max*	0–100	0–100	0–100	

*M*—mean; *SD*—standard deviation; *Me*—median; *Q*_1_—lower quartile; *Q*_3_—upper quartile; *Min*—lowest value; *Max*—highest value.

**Table 7:** Quality of life (EORTC QLQ-30C scores) in patients differing in terms of scores for the destructive coping strategy (Mini-MAC), with analysis of variance results.

QoL Evaluation—EORTC QLQ-30C	Destructive Coping Strategy (Mini-MAC)			ANOVA *p*
	Low Score Sten 0–4	Moderate Score Sten 5–6	High Score Sten 7–10	
	*n* = 126	*n* = 59	*n* = 17	
General health (QL)				<0.001
*M* ± *SD*	64.0 ± 17.5	55.7 ± 16.6	22.5 ± 18.8	
*Me* (*Q*_1_; *Q*_3_)	67 (50; 81)	58 (50; 67)	17 (8; 33)	
*Min–Max*	17–100	8–83	0–58	
Physical functioning (PF)				<0.001
*M* ± *SD*	60.6 ± 16.3	20.5 ± 12.3	20.8 ± 13.5	
*Me* (*Q*_1_; *Q*_3_)	60 (53; 73)	20 (13; 27)	20 (13; 27)	
*Min–Max*	20–87	0–60	0–60	
Role functioning (RF)				<0.001
*M* ± *SD*	60.8 ± 22.0	15.3 ± 15.3	16.5 ± 17.9	
*Me* (*Q*_1_; *Q*_3_)	67 (50; 67)	17 (0; 17)	17 (0; 33)	
*Min–Max*	17–100	0–67	0–67	
Emotional functioning (EF)				<0.001
*M* ± *SD*	71.6 ± 14.5	44.8 ± 24.2	29.7 ± 20.5	
*Me* (*Q*_1_; *Q*_3_)	75 (67; 83)	42 (25; 67)	25 (17; 33)	
*Min–Max*	42–92	0–100	0–92	
Cognitive functioning (CF)				<0.001
*M* ± *SD*	63.7 ± 23.7	25.1 ± 22.8	19.3 ± 21.4	
*Me* (*Q*_1_; *Q*_3_)	67 (50; 83)	17 (0; 33)	17 (0; 33)	
*Min–Max*	33–100	0–100	0–83	
Social functioning (SF)				<0.001
*M* ± *SD*	63.7 ± 18.9	37.9 ± 24.7	20.6 ± 18.4	
*Me* (*Q*_1_; *Q*_3_)	67 (50; 83)	33 (25; 50)	17 (0; 33)	
*Min–Max*	33–100	0–100	0–83	
Fatigue (FA)				<0.001
*M* ± *SD*	37.5 ± 21.8	38.4 ± 20.5	62.1 ± 16.7	
*Me* (*Q*_1_; *Q*_3_)	33 (22; 53)	33 (22; 56)	67 (44; 78)	
*Min–Max*	0–100	0–100	33–89	
Nausea and vomiting (NV)				<0.001
*M* ± *SD*	8.1 ± 19.4	14.7 ± 22.5	64.7 ± 26.3	
*Me* (*Q*_1_; *Q*_3_)	0 (0; 0)	0 (0; 33)	67 (50; 83)	
*Min–Max*	0–100	0–100	17–100	
Pain (PA)				<0.001
*M* ± *SD*	23.8 ± 23.0	28.2 ± 24.0	61.8 ± 20.2	
*Me* (*Q*_1_; *Q*_3_)	17 (0; 33)	33 (17; 33)	67 (50; 67)	
*Min–Max*	0–100	0–100	17–100	
Dyspnea (DY)				<0.001
*M* ± *SD*	11.6 ± 21.6	7.9 ± 18.9	60.4 ± 30.4	
*Me* (*Q*_1_; *Q*_3_)	0 (0; 33)	0 (0; 0)	67 (58; 67)	
*Min–Max*	0–100	0–67	0–100	
Insomnia (SL)				<0.001
*M* ± *SD*	35.7 ± 34.0	42.0 ± 30.9	70.6 ± 20.0	
*Me* (*Q*_1_; *Q*_3_)	33 (0; 67)	33 (33; 67)	67 (67; 67)	
*Min–Max*	0–100	0–100	33–100	
Appetite loss (AP)				<0.001
*M* ± *SD*	13.2 ± 21.1	23.7 ± 30.4	66.7 ± 16.7	
*Me* (*Q*_1_; *Q*_3_)	0 (0; 33)	0 (0; 33)	67 (67; 67)	
*Min–Max*	0–100	0–100	33–100	
Constipation (CO)				<0.001
*M* ± *SD*	17.2 ± 24.1	19.8 ± 21.5	51.0 ± 20.8	
*Me* (*Q*_1_; *Q*_3_)	0 (0; 33)	33 (0; 33)	67 (33; 67)	
*Min–Max*	0–100	0–67	0–67	
Diarrhea (DI)				<0.001
*M* ± *SD*	6.9 ± 17.5	7.9 ± 19.9	51.0 ± 29.1	
*Me* (*Q*_1_; *Q*_3_)	0 (0; 0)	0 (0; 0)	67 (33; 67)	
*Min–Max*	0–100	0–100	0–100	
Financial difficulties (FI)				<0.001
*M* ± *SD*	22.8 ± 24.8	34.5 ± 30.6	68.6 ± 32.2	
*Me* (*Q*_1_; *Q*_3_)	33 (0; 33)	33 (0; 58)	67 (33; 100)	
*Min–Max*	0–100	0–100	0–100	

*M*—mean; *SD*—standard deviation; *Me*—median; *Q*_1_—lower quartile; *Q*_3_—upper quartile; *Min*—lowest value; *Max*—highest value.

**Table 8:** Quality of life (EORTC QLQ-BR23 scores) in patients differing in terms of scores for the destructive coping strategy, with analysis of variance results.

Functioning Scales QLQ-BR23	Destructive Coping Strategy (Mini-MAC)			ANOVA *p*
	Low Score Sten 0–4	Moderate Score Sten 5–6	High Score Sten 7–10	
	*n* = 126	*n* = 59	*n* = 17	
Body image (BRBI)				<0.001
*M* ± *SD*	64.2 ± 13.4	52.1 ± 29.0	29.5 ± 24.3	
*Me* (*Q*_1_; *Q*_3_)	67 (58; 75)	50 (33; 75)	25 (8; 42)	
*Min–Max*	33–83	0–100	0–100	
Sexual functioning (BRSEF)				<0.001
*M* ± *SD*	64.7 ± 16.5	49.7 ± 32.1	27.2 ± 26.6	
*Me* (*Q*_1_; *Q*_3_)	67 (50; 83)	50 (33; 67)	33 (0; 33)	
*Min–Max*	33–83	0–100	0–100	
Sexual enjoyment (BRSEE)				0.016
*M* ± *SD*	44.0 ± 37.1	37.3 ± 32.1	17.6 ± 29.1	
*Me* (*Q*_1_; *Q*_3_)	33 (0; 67)	33 (0; 67)	0 (0; 33)	
*Min–Max*	0–100	0–100	0–100	
Future outlook (BRFU)				<0.001
*M* ± *SD*	72.6 ± 27.0	86.4 ± 24.9	65.3 ± 30.9	
*Me* (*Q*_1_; *Q*_3_)	67 (67; 100)	100 (83; 100)	67 (33; 100)	
*Min–Max*	33–100	33–100	0–100	
Systemic treatment adverse effects (BRST)				<0.001
*M* ± *SD*	23.3 ± 16.4	33.5 ± 19.7	58.8 ± 12.5	
*Me* (*Q*_1_; *Q*_3_)	19 (10; 33)	29 (19; 48)	62 (52; 67)	
*Min–Max*	0–71	0–100	33–76	
Breast-related symptoms (BRBS)				<0.001
*M* ± *SD*	20.0 ± 22.7	34.1 ± 21.6	62.9 ± 19.7	
*Me* (*Q*_1_; *Q*_3_)	8 (0; 33)	33 (17; 50)	58 (50; 75)	
*Min–Max*	0–92	0–75	25–92	
Arm-related symptoms (BRAS)				<0.001
*M* ± *SD*	28.2 ± 21.4	43.1 ± 24.7	62.1 ± 16.2	
*Me* (*Q*_1_; *Q*_3_)	22 (11; 44)	44 (22; 67)	56 (56; 78)	
*Min–Max*	0–100	0–89	33–89	
Concern about hair loss (BRHL)				<0.001
*M* ± *SD*	40.9 ± 35.7	70.2 ± 30.8	60.8 ± 24.3	
*Me* (*Q*_1_; *Q*_3_)	33 (0; 67)	67 (67; 100)	67 (67; 67)	
*Min–Max*	0–100	0–100	0–100	

*M*—mean; *SD*—standard deviation; *Me*—median; *Q*_1_—lower quartile; *Q*_3_—upper quartile; *Min*—lowest value; *Max*—highest value.

**Table 9:** Quality of life (EORTC QLQ-BR23 scores) in patients differing in terms of pain severity scores, with analysis of variance results.

QoL Evaluation—EORTC QLQ-30C	Pain Severity (VAS)			ANOVA *p*
	Mild 0–2 Points	Moderate 2–4 Points	Severe 4–10 Points	
	*n* = 100	*n* = 52	*n* = 50	
General health (QL)				<0.001
*M* ± *SD*	68.3 ± 13.5	58.3 ± 16.3	37.2 ± 21.3	
*Me* (*Q*_1_; *Q*_3_)	67 (58; 83)	58 (50; 67)	33 (25; 50)	
*Min*–*Max*	33–100	17–100	0–83	
Physical functioning (PF)				<0.001
*M* ± *SD*	41.7 ± 20.3	22.5 ± 11.2	16.0 ± 11.0	
*Me* (*Q*_1_; *Q*_3_)	40 (27; 58)	20 (13; 27)	13 (7; 20)	
*Min*–*Max*	7–87	7–53	0–47	
Role functioning (RF)				<0.001
*M* ± *SD*	43.0 ± 23.4	19.9 ± 14.8	8.3 ± 12.2	
*Me* (*Q*_1_; *Q*_3_)	50 (17; 62)	17 (17; 33)	0 (0; 17)	
*Min*–*Max*	0–100	0–67	0–67	
Emotional functioning (EF)				<0.001
*M* ± *SD*	60.0 ± 20.9	42.6 ± 21.1	23.9 ± 17.6	
*Me* (*Q*_1_; *Q*_3_)	67 (42; 75)	33 (31; 58)	25 (17; 33)	
*Min*–*Max*	17–100	0–83	0–92	
Cognitive functioning (CF)				<0.001
*M* ± *SD*	46.0 ± 29.1	25.6 ± 20.5	13.7 ± 17.0	
*Me* (*Q*_1_; *Q*_3_)	33 (33; 67)	33 (0; 33)	17 (0; 17)	
*Min*–*Max*	0–100	0–83	0–83	
Social functioning (SF)				<0.001
*M* ± *SD*	49.0 ± 24.8	30.4 ± 21.8	18.8 ± 18.1	
*Me* (*Q*_1_; *Q*_3_)	50 (33; 67)	33 (17; 37)	17 (0; 33)	
*Min*–*Max*	0–100	0–67	0–100	

*M*—mean; *SD*—standard deviation; *Me*—median; *Q*_1_—lower quartile; *Q*_3_—upper quartile; *Min*—lowest value; *Max*—highest value.

**Table 10:** Quality of life (EORTC QLQ-BR23 scores) in patients differing in terms of pain severity scores, with analysis of variance results.

Functioning Scales QLQ-BR23	Pain Severity (VAS)			ANOVA *p*
	Mild 0–2 Points	Moderate 2–4 Points	Severe 4–10 Points	
	*n* = 100	*n* = 52	*n* = 50	
Body image (BRBI)				<0.001
*M* ± *SD*	46.2 ± 28.3	47.1 ± 30.1	31.3 ± 24.7	
*Me* (*Q*_1_; *Q*_3_)	46 (25; 67)	33 (33; 75)	33 (8; 50)	
*Min*–*Max*	0–100	0–100	0–100	
Sexual functioning (BRSEF)				<0.001
*M* ± *SD*	46.3 ± 28.2	45.2 ± 33.4	28.0 ± 27.6	
*Me* (*Q*_1_; *Q*_3_)	50 (21; 67)	33 (33; 67)	33 (0; 33)	
*Min*–*Max*	0–100	0–100	0–100	
Sexual enjoyment (BRSEE)				0.021
*M* ± *SD*	41.0 ± 33.5	47.7 ± 35.5	27.0 ± 37.0	
*Me* (*Q*_1_; *Q*_3_)	33 (0; 67)	33 (33; 67)	0 (0; 67)	
*Min*–*Max*	0–100	0–100	0–100	
Future outlook (BRFU)				0.023
*M* ± *SD*	80.7 ± 27.0	74.4 ± 30.0	66.7 ± 31.2	
*Me* (*Q*_1_; *Q*_3_)	100(67;100)	100(33;100)	67 (33; 100)	
*Min*–*Max*	0–100	0–100	0–100	
Systemic treatment adverse effects (BRST)				<0.001
*M* ± *SD*	21.0 ± 17.5	30.8 ± 17.7	44.3 ± 17.4	
*Me* (*Q*_1_; *Q*_3_)	14 (10; 29)	29 (14; 43)	43 (33; 57)	
*Min*–*Max*	0–100	0–67	5–76	
Breast-related symptoms (BRBS)				<0.001
*M* ± *SD*	16.7 ± 19.4	28.9 ± 20.9	48.7 ± 26.5	
*Me* (*Q*_1_; *Q*_3_)	8 (0; 25)	33 (8; 42)	50 (33; 73)	
*Min*–*Max*	0–92	0–67	0–92	
Arm-related symptoms (BRAS)				<0.001
*M* ± *SD*	28.1 ± 21.1	39.5 ± 24.5	45.8 ± 25.9	
*Me* (*Q*_1_; *Q*_3_)	22 (11; 44)	44 (22; 56)	50 (22; 67)	
*Min*–*Max*	0–89	0–100	0–89	
Concern about hair loss (BRHL)				0.642
*M* ± *SD*	51.1 ± 34.5	52.7 ± 38.3	58.3 ± 33.2	
*Me* (*Q*_1_; *Q*_3_)	67 (33; 67)	67 (33; 100)	67 (33; 75)	
*Min*–*Max*	0–100	0–100	0–100	

*M*—mean; *SD*—standard deviation; *Me*—median; *Q*_1_—lower quartile; *Q*_3_—upper quartile; *Min*—lowest value; *Max*—highest value.

## Data Availability

All the data analyzed during the current study are available from the corresponding author on reasonable request.
